# Description of three new species of oak gallwasps of the genus *Amphibolips* Reinhard from Mexico (Hymenoptera, Cynipidae)

**DOI:** 10.3897/zookeys.987.51366

**Published:** 2020-11-06

**Authors:** Dohuglas Eliseo Castillejos-Lemus, Ken Oyama, José Luis Nieves-Aldrey

**Affiliations:** 1 Escuela Nacional de Estudios Superiores (ENES) Unidad Morelia, Universidad Nacional Autónoma de México (UNAM). Antigua Carretera a Pátzcuaro 8701, Ex-Hacienda de San José de la Huerta, 58190, Morelia, Michoacán, México Universidad Nacional Autónoma de México Morelia Mexico; 2 Departamento de Biodiversidad y Biología Evolutiva, Museo Nacional de Ciencias Naturales (CSIC). José Gutiérrez Abascal 2, 28006 Madrid, Spain Museo Nacional de Ciencias Naturales Madrid Spain

**Keywords:** *
Amphibolips
*, Cynipini, *
Lobatae
*, Mexico, oak apple gall, oak gallwasps, *
Quercus
*

## Abstract

Three new species of oak gall wasps of the genus *Amphibolips* Reinhard, 1865 (Hymenoptera: Cynipidae: Cynipini) are described from Mexico: *Amphibolips magnigalla* Nieves-Aldrey & Castillejos-Lemus, *Amphibolips kinseyi* Nieves-Aldrey & Castillejos-Lemus and *Amphibolips nigrialatus* Nieves-Aldrey & Castillejos-Lemus. The specimens of the first two species were representative of sexual generations and come from the State of Oaxaca, while only a female, collected in the State of Veracruz, is described for *A. nigrialatus*. The new species induces galls on *Quercus zempoaltepecana* and *Q. sapotifolia* (Fagaceae, section Lobatae, red oaks). Descriptions of the diagnostic morphological characteristics of the three species and a key for their identification are provided. The taxonomic relationships of the new species with other species of *Amphibolips* are discussed; the three new species are closely allied amongst themselves and are related to *A. dampfi* Kinsey, 1937. With the three newly-described species, the number of *Amphibolips* in Mexico is increased to 23.

## Introduction

Oak gall wasps (Cynipidae: Cynipini) include approximately 41 genera with circa 1,000 species (Liljeblad et al. 2008, Pénzes et al. 2018) distributed mainly in the Holarctic, Neotropical and Oriental regions (Ronquist et al. 2015). They represent the largest tribe of Cynipidae and are a monophyletic group of wasps that induce relatively more structurally complex and diverse galls of the known gall types (Cornell 1983, Stone and Cook 1998, Nieves-Aldrey 2001, Csöka et al. 2005). Cynipini species are predominantly associated with host species of *Quercus* (Liljeblad et al. 2008), but some genera of Cynipini use other hosts within the Fagaceae, such as *Castanea*, *Castanopsis*, *Lithocarpus*, *Chrysolepis* and *Notholithocarpus* (Stone et al. 2002, Nicholls et al. 2018). A particularity of the Cynipini is that most species exhibit life histories with alternating generations (e.g. asexual and sexual) (Stone et al. 2002).

The Nearctic region, particularly Mexico, is one of the centres of diversity of the oak gall wasps, which have been estimated to include more than 700 species (Stone et al. 2002, Liu et al. 2007). This diversity is directly related to the diversity of *Quercus* species, with more than 90 species recorded from the United States and Canada and 161 from Mexico (Valencia 2004, Liu and Ronquist 2006). The most recent work on the number of Cynipidae species recorded from Mexico indicates the presence of 183 species in 16 genera (Pujade-Villar and Ferrer-Suay 2015) associated with approximately 35 *Quercus* species.

*Amphibolips* is exclusively associated with the Lobatae section of Quercus genus and is restricted to the American continent (Nieves-Aldrey et al. 2012). Fifty-three species of *Amphibolips* are recognised; the vast majority are found in the Nearctic region, three species are distributed in Panama, 19 species are endemic to Mexico and one is shared with the United States (Burks 1979, Melika and Abrahamson 2002, Medianero and Nieves-Aldrey 2010, Melika et al. 2011, Nieves-Aldrey et al. 2012, Pujade-Villar et al. 2018).

The morphological characteristics of adults and their galls are very uniform amongst the most well-known *Amphibolips* species. The galls induced by species of this genus develop mainly in buds, stems or leaves and are rarely found in acorns. They are usually globose or spindle-shaped and detachable, with a spongy parenchyma surrounding a central larval cell, sometimes supported by radiating filaments (Beutenmüller 1909, Kinsey 1937, Melika and Abrahamson 2002). The species of this genus can be easily recognised by the following diagnostic characteristics: antennae with 12 to 14 segments in females and 15 to 16 segments in males; body robust with strong coarse reticulate sculpture, notauli not well marked, mesoscutellum often emarginate posteriorly; metasomal tergites punctate posteriorly; metatarsal claws with a large secondary basal tooth; forewings usually more or less smoky and showing spots, bands or completely obscured; radial cell open; ventral spine of the hypopygium usually long and pointed apically, without setae forming an apical tuft (Melika and Abrahamson 2002, Medianero and Nieves-Aldrey 2010, Melika et al. 2011).

Before 1937, only two species had been described in Mexico (*A. palmeri* Basset, 1890 and *A. nigra* Beutenmüller, 1911) (Bassett 1890; Beutenmüller 1911, 1917). In 1937, Kinsey described nine species, six of which he grouped in the “*niger*” complex; the remaining three (*A. dampfi* Kinsey, 1937, *A. nassa* Kinsey, 1937 and *A. fusus* Kinsey, 1937) were not grouped. Melika et al. (2011) described two new species: *A. zacatecaensis* Melika & Pujade-Villar, 2011 and *A. hidalgoensis* Pujade-Villar & Melika, 2011. Parallel to the “*niger*” complex proposed by Kinsey (1937), a second group, the “*nassa*” species complex, was proposed and a species identification key was provided for *A. palmeri*, *A. dampfi*, *A. nassa*, *A. hidalgoensis*, *A. zacatecaensis* and *A. fusus*. Nieves-Aldrey et al. (2012) described seven new species outside of those in the “*niger*” group, raising the number of known species to 13. In the referenced paper, the “*nassa*” complex was criticised as useless, based on the assumption that it did not reflect the extant species diversity outside of the “*niger*” group, as the complex omitted the anterior wing colouration pattern, which was important for some of the species described by Kinsey, such as *A. dampfi*, *A. fusus* and *A. nassa* (Nieves-Aldrey et al. 2012). More recently, an additional species (*A. cibriani* Pujade-Villar, 2018) was described within the “*nassa*” group (Pujade-Villar et al. 2018), resulting in a total of 20 species of *Amphibolips* recorded from Mexico.

The objective of this study is to present a description of three new species of the *Amphibolips* genus in Mexico. One of these species represents the first record of *Amphibolips* for the State of Veracruz (*A. nigrialatus* Nieves-Aldrey & Castillejos-Lemus, new species) and the other two species were collected in the State of Oaxaca. One of the species from Oaxaca induces a strikingly-characteristic gall (*A. magnigalla* Nieves-Aldrey & Castillejos-Lemus, new species), while the other species from Oaxaca (*A. kinseyi* Nieves-Aldrey & Castillejos-Lemus, new species) shares characteristics with *A. magnigalla* and with another species previously described from Oaxaca (*A. dampfi* Kinsey, 1937). The species richness of this genus in Mexico is discussed, as well as the taxonomic problems existing within the group. An update to the identification key given in Nieves-Aldrey et al. (2012) is provided, including the new species described herein. This work is part of a larger study of the revision of the *Amphibolips* species of Mexico, which includes extensive sampling throughout most of Mexico. Rich materials of *Amphibolips* have been collected, including possible additional new species and are being studied with a phylogenetic approach, including genetic tools. The results will be published elsewhere.

## Material and methods

### Study material

*Quercus* species of the Lobatae section were sampled in Veracruz State in 2008 and in Oaxaca in 2018. The galls were collected directly from oak trees and stored in plastic containers with plastic or mesh lids until the emergence of the wasps. The emergence of the wasps occurred under laboratory conditions. The voucher specimens and their galls were deposited in the entomological collections of the Museo Nacional de Ciencias Naturales in Madrid, Spain and in the Colección Nacional de Insectos of the Instituto de Biología, Universidad Nacional Autónoma de México, Mexico City, Mexico. The *Quercus* species were identified by Dr Susana Valencia-Ávalos at the Facultad de Ciencias of the Universidad Nacional Autónoma de México (UNAM). Voucher specimens were deposited in the Herbarium of the Facultad de Ciencias and in the Escuela Nacional de Estudios Superiores, Unidad Morelia (ENES-Morelia) of the UNAM. Observations on the habitats, distribution or affinities of the host *Quercus* species were mainly based on Valencia-Ávalos (2004), but other publications on *Quercus* species were also consulted.

### Examination of types

The type specimen of *Amphibolips dampfi* Kinsey, 1937 was examined for comparison with the new species described. The male holotype of this species was borrowed from the American Museum of Natural History, New York (AMNH) (James Carpenter).

### Specimen preparation

The images used for the morphological descriptions were taken with a FEI Quanta 200 (Oregon, EU) scanning electron microscope (SEM) in Madrid (Spain) and with a JEOL JSM-IT300 (Tokyo, Japan) SEM in Morelia (Mexico). For the SEM observations, two strategies were followed, depending on the number of individuals available for a given species. For the preservation of some unique specimens mounted in a conventional manner, a low vacuum technique was used without gold coating. When the number of specimens allowed it, some specimens were dissected in 99% alcohol and mounted in stubs to be coated with gold and observed with a high vacuum technique. The forewings were mounted on slides with euparal and examined with a Wild MZ8 and an Olympus SZX10 stereomicroscope. Images of the wings and adult habitus were acquired with a NIKON Coolpix 4500 digital camera attached to a Wild MZ8 light microscope, with the exception of some images taken with an Olympus SC100 camera with the help of CELLSENS STANDARD software. Measurements were made with a micrometric eyepiece calibrated to a Wild M5A stereomicroscope. Photographs of galls in the field and of gall dissections were taken with a Nikon D5300 camera.

### Morphological terms

The terminology of the morphological structures and abbreviations follow that of Ronquist and Nordlander (1989), Nieves-Aldrey (2001) and Liljeblad et al. (2008). For wing venation, we follow Ronquist and Nordlander (1989) and for the terminology of the forewing cells, we follow Richards and Davies (1977). For sculpture terminology, we follow Harris (1979). The measurements of the structures were made according to Nieves-Aldrey (2001). The abbreviations used include F1–F12 for the antennal flagellomeres, **POL** (post-ocellar distance) for the distance between the inner margins of the posterior ocelli, **OOL** (ocellar-ocular distance) for the distance from the outer margin of a posterior ocellus to the inner margin of a compound eye and **DOL** (diameter of a lateral ocellus).

## Results

### Amphibolips magnigalla

Taxon classificationAnimaliaHymenopteraCynipidae

Nieves-Aldrey & Castillejos-Lemus sp. nov.

F75CFB16-90BA-56BF-9408-9389D6CDAF7D

http://zoobank.org/34858B85-8A3C-4675-9ADD-76BABA811ABA

**Figure 1.**
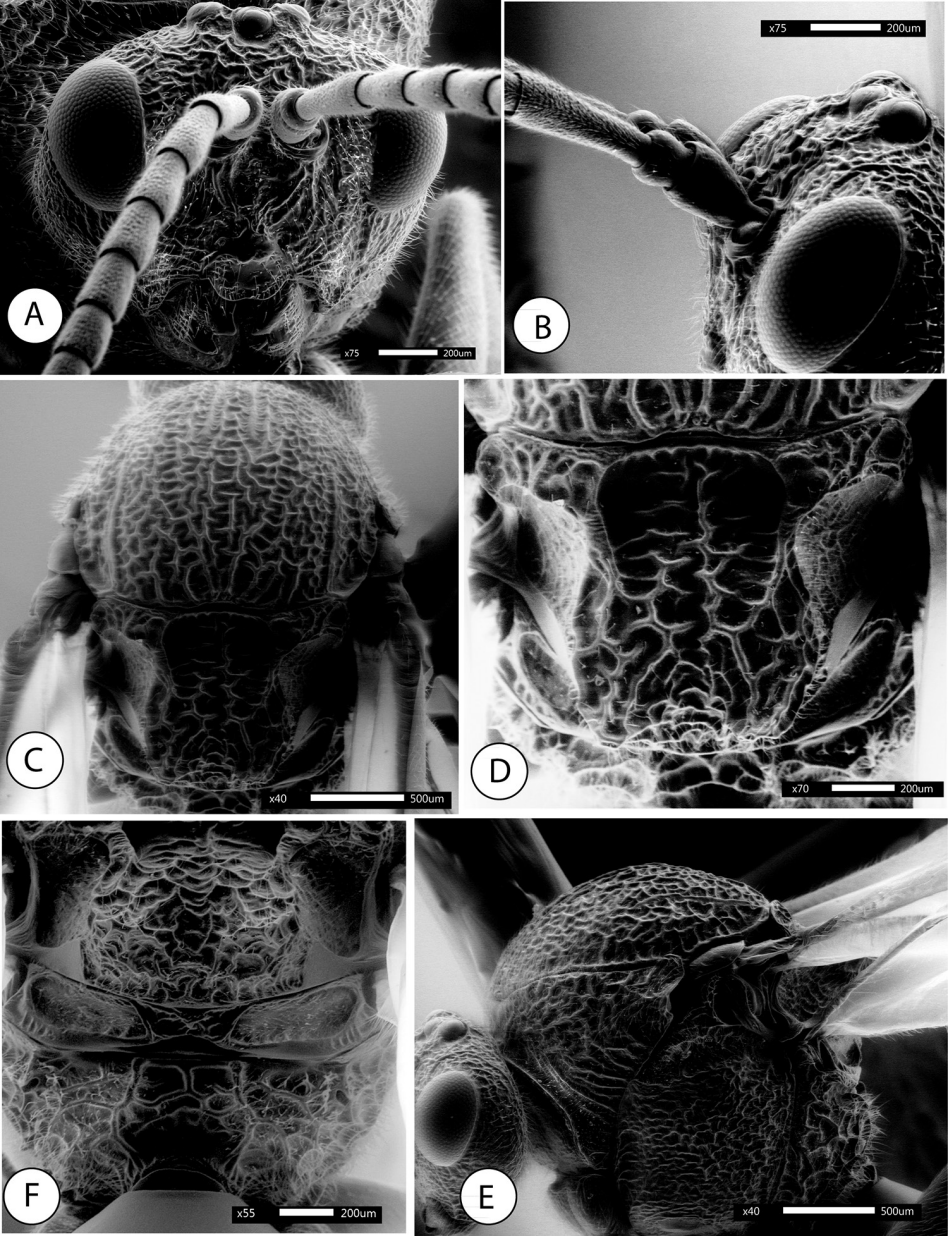
*Amphibolips magnigalla* sp. nov., female **A** head, anterior view **B** frons and vertex **C** mesosoma, dorsal view **D** scutellum, dorsal view **E** mesosoma, lateral view **F** propodeum.

**Figure 2.**
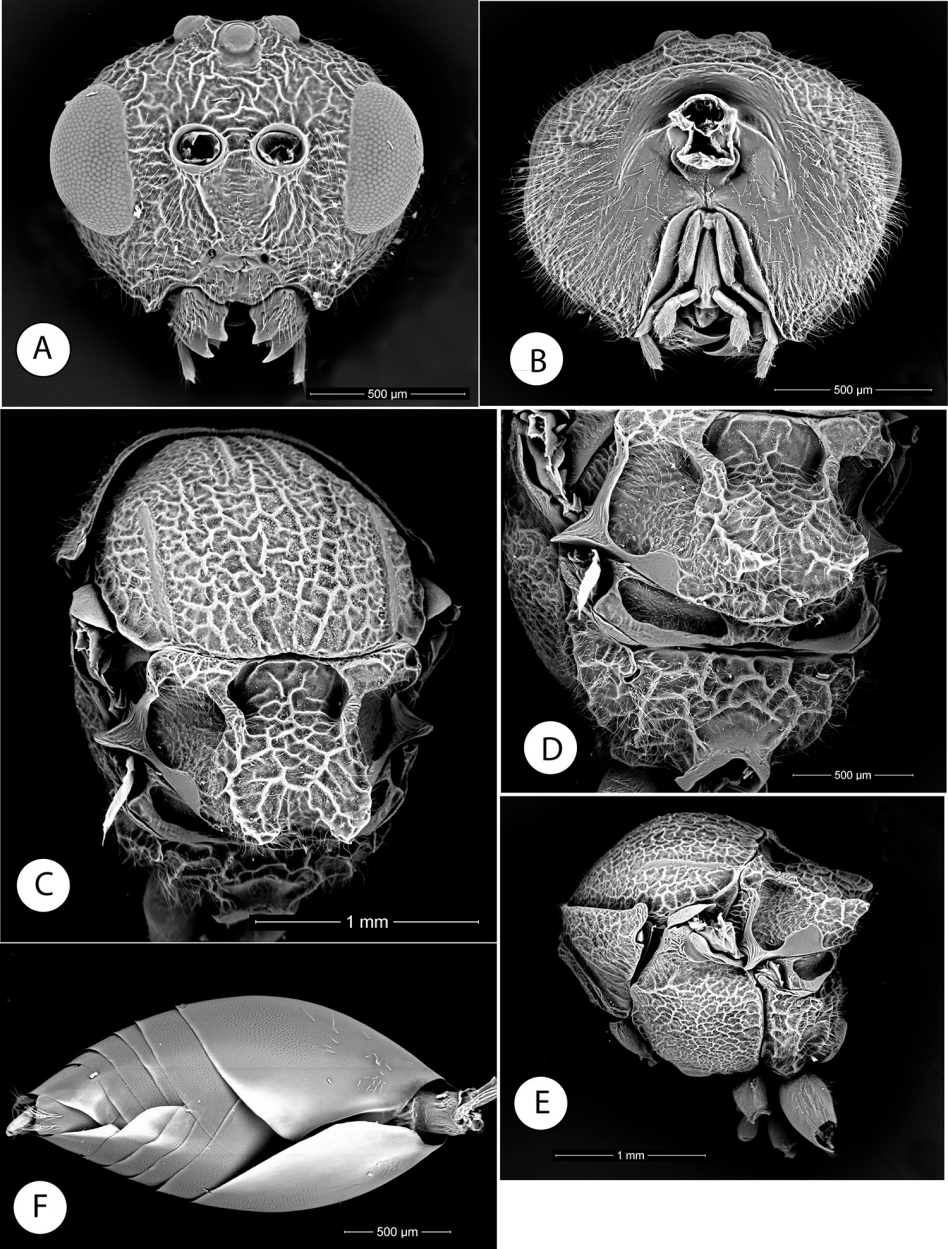
*Amphibolips magnigalla* sp. nov., male **A** head, anterior view **B** head, posterior view **C** mesosoma, dorsal view **D** scutellum and propodeum **E** mesosoma, lateral view **F** metasoma, ventral view.

**Figure 3.**
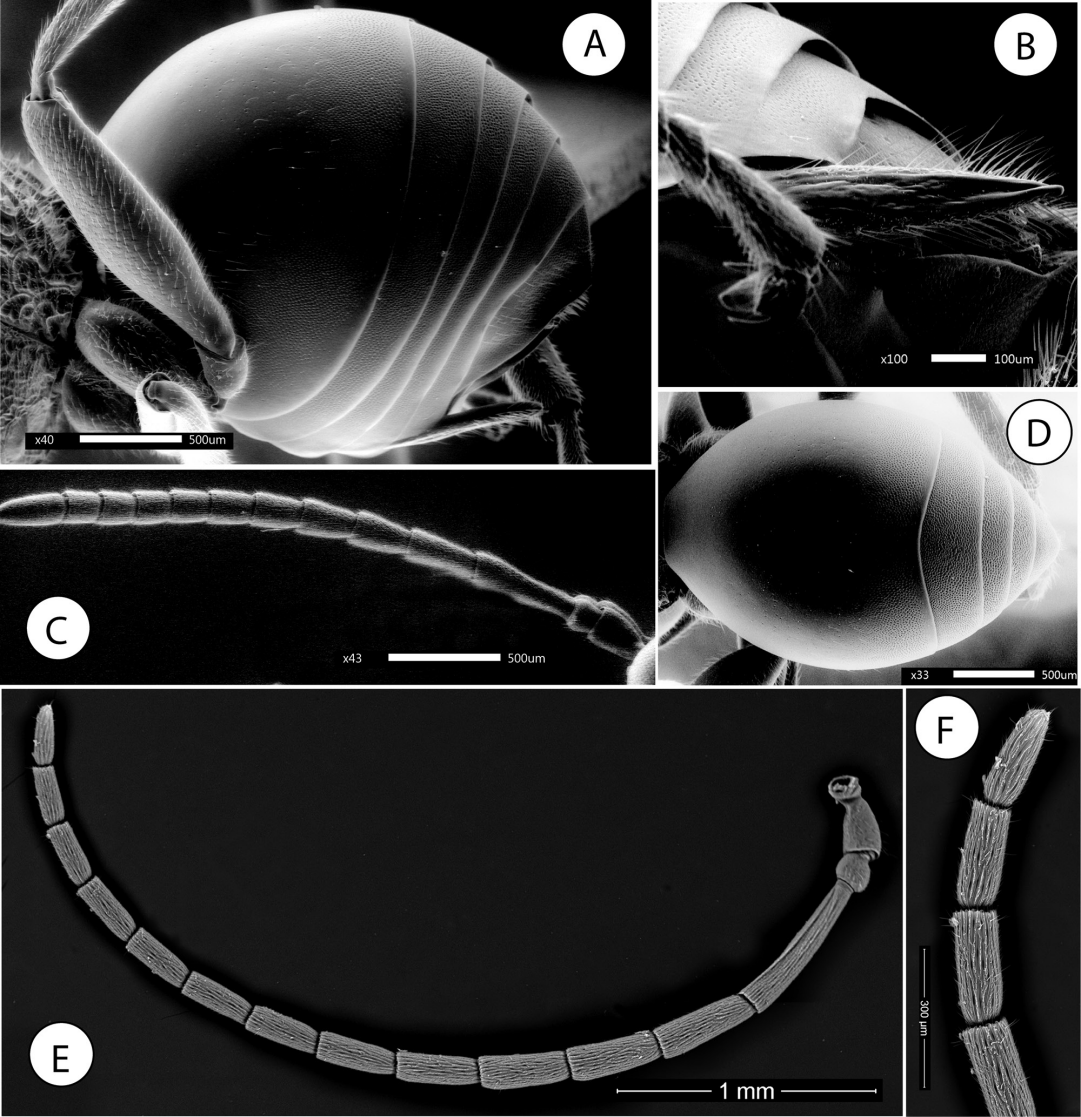
*Amphibolips magnigalla* sp. nov. **A** female metasoma, lateral view **B** female hypopygium, ventral view **C** female antenna **D** female metasoma, dorsal view **E** male antenna **F** detail of apical flagellomeres in male antenna.

**Figure 4.**
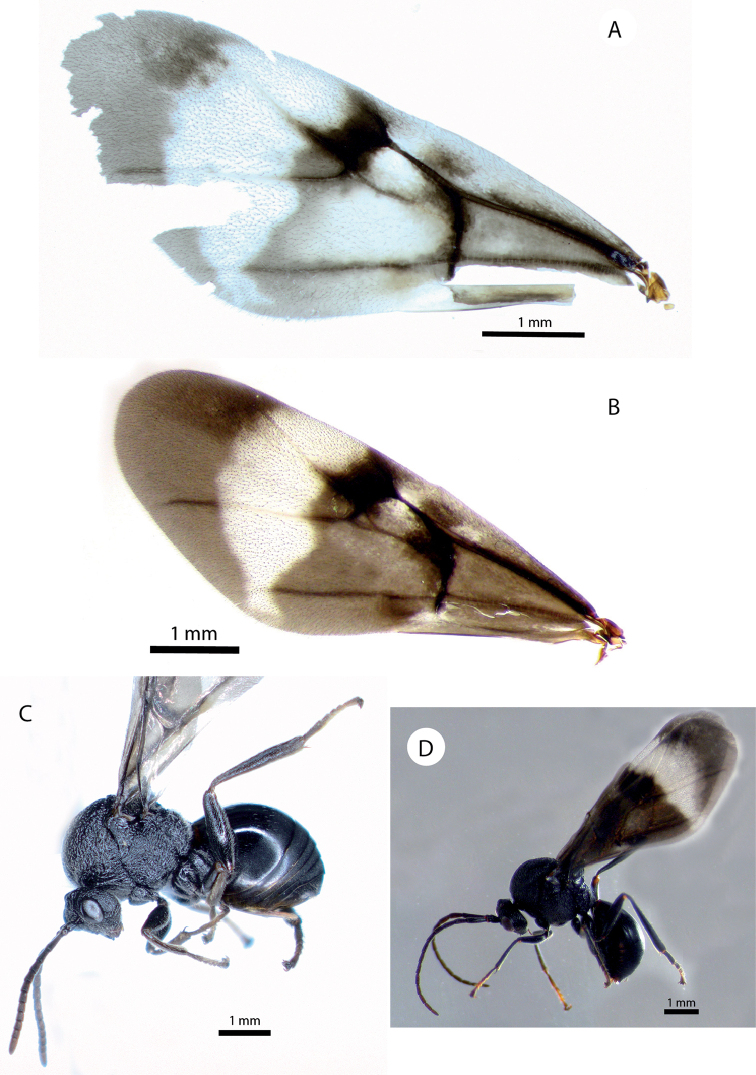
*Amphibolips magnigalla* sp. nov. **A** female forewing **B** male forewing **C** female habitus **D** male habitus.

**Figure 5.**
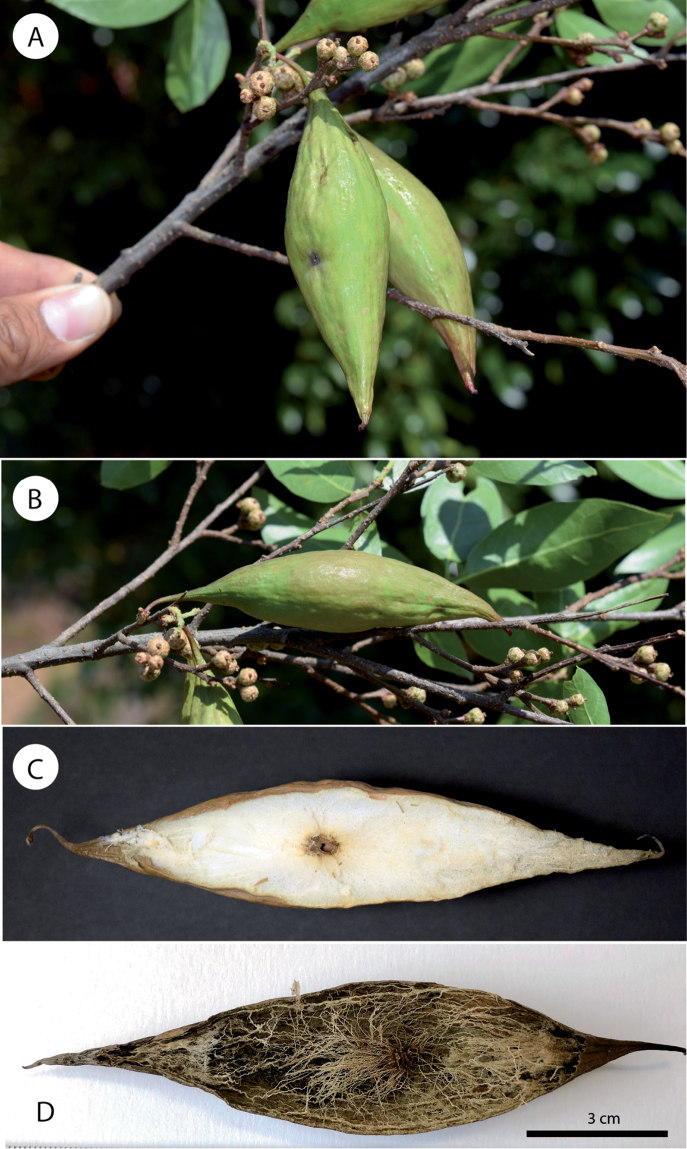
*Amphibolips magnigalla* sp. nov. **A–B** galls **C–D** sections of galls.

Figs 1 , 2 , 3 , 4 , 5

#### Type material.

***Holotype***: 1♀ in the Museo Nacional de Ciencias Naturales, Madrid, Spain (MNCN), mounted (glued) on a card. Mexico, Oaxaca, Comaltepec, 17°33'50"N, 96°33'20"W, ca. 2330 m alt.; ex gall *Quercus zempoaltepecana* (Quercus sect. Lobatae), gall collected 21/04/2018, insect emerged 30/04/2018. D. Castillejos-Lemus leg. ***Paratypes***: 5♂, same data as holotype but emerged 1-3/05/2018. Two paratype ♂ were dissected and mounted on a stub for SEM observation in the MNCN. Other materials: 4♂, same data as paratypes, preserved in ethanol (in MNCN and Castillejos-Lemus collection, Morelia, Mexico). Additional material: 3 galls, one dissected (in MNCN).

#### Etymology.

Named after the strikingly-large size of the galls of this species.

#### Diagnosis and comments.

This new species belongs to the group of *Amphibolips* species that have a forewing with a transversal clear band that is variable in size and extends towards medial and cubital veins to the ventral margin of the wing (Nieves-Aldrey et al. 2012). The aforementioned group comprises *Amphibolips castroviejoi* from Panama, *Amphibolips trizonata* Ashmead, 1896 from Arizona (USA) and the Mexican *Amphibolips durangensis* Nieves-Aldrey & Maldonado, 2012, *A. dampfi* and the recently described *A. cibriani* Pujade-Villar, 2018 (Pujade-Villar et al. 2018). However, the forewing pattern of the new species is different from that of all the referenced species. The transversal clear band is larger and broader and extends to two-thirds of the radial cell in both sexes and the basal third of the wing is more heavily infuscate in the male (Fig. 4A, B).

*Amphibolips magnigalla* shares with *Amphibolips dampfi*, *A. castroviejoi* and the other two new species described herein, a mesoscutellum emarginate posteriorly. However, the emargination is comparatively less deep in *A. magnigalla* (Figs 1C, 2C). Besides the main character of the forewing, the four species can be readily distinguished by the characters provided in the identification key in this paper.

Regarding the gall, the new species is easily distinguished by its large spindle-shaped gall (approximately 10 cm in length × 2.5 cm in diameter), which is at least 2× larger than any other spindle-shaped gall described from Mexico. *Amphibolips fusus* and *A. durangensis* induce galls morphologically similar to the gall of the new species. However, besides the differences in size, the inner structure of the gall induced by the referenced species is different, being filled with a dense soft tissue, while the inner structure of the gall induced by the new species is often almost empty, with visible radiating filaments from the central larval cell.

#### Description.

Body length: 5.8 mm (n = 1) for females; 5.2 mm (n = 3) for males.

Female (Fig. 4C). Body almost entirely black; antennae, except two basal segments, mandibles, metasoma ventrally, hypopygium and parts of tibiae and tarsi, chestnut. Forewing predominantly black infuscate, except a wide clear transversal band that starts in the distal two thirds of radial cell and extends towards discoidal and cubital cell, almost reaching ventral margin of forewing. Another non-infuscate band extended from the posterior part of the costal cell towards the Rs+M vein and reached the cubital vein (Fig. 4A).

Head, in dorsal view 2.3× wider than long. POL:OOL:DOL as 23:44:14. Head in anterior view (Fig. 1A) 1.2× wider than high, gena slightly broadened behind eye. Vertex, frons, lower face, gena and occiput with strong reticulate-rugose sculpture (Fig. 1B); two longitudinal carina present, extending from ventral margin of toruli to converge towards the anterior tentorial pits; irradiating carinae from clypeus absent; head moderately pubescent, except in vertex and frons. Clypeus more or less hexagonal, ventral margin strongly projecting over mandibles and slightly sinuate on anterior margin. Anterior tentorial pits well visible; epistomal sulcus and clypeo-pleurostomal lines slightly discernible. Malar space 0.7× height of compound eye. Toruli situated mid-height of compound eye; transfacial line 1.4× height of an eye; distance between antennal rim and compound eye slightly shorter than width of antennal socket including rim. Ocellar plate slightly raised.

Head posterior view (male) (Fig. 2B), heavily pubescent, with occiput coarsely rugose; dorsally the sculpture is transversely ribbed. Two carinae present, arising from dorsal part of the occipital foramen and ventrally continuing past posterior tentorial pits; posterior tentorial pits rounded; gular sulci united meeting at hypostoma. Posterodorsal margin of oral foramen not margined medially; hypostomal ridges well separated.

Mouthparts (male) (Fig. 2A), mandibles strong, exposed; with dense setae in base, right mandible with three teeth; left with two teeth. Cardo of maxilla not visible, maxillary stipes 4.1× as long as wide. Maxillary palp with five segments. Labial palp with three segments; apical peg of last labial and maxillary segments present.

Antenna (Fig. 3C), of moderate length, 0.5× as long as body length; with 13 antennomeres; flagellum not broadening towards apex; with relatively long, erect setae. Relative length/width of antennal segments as: 0.29(0.16):0.12(0.16):0.44(0.15):0.32(0.14):0.25(0.15):0.24(0.16):0.21(0.15):0.19(0.15): 0.17(0.14): 0.16(0.15):0.16(0.15):0.16(0.15):0.32(0.14). Pedicel (Fig. 3C) short, small, broader than long, 0.4× as long as scape; F1 1.4× as long as F2. F8-F10 as long as wide, F11 2.3× as long as wide, 2× as long as F10. Placodeal sensilla on F5-F11, disposed in dense rows of 6–8 sensilla, only in half dorsal area of each flagellomere.

Mesosoma in lateral view (Fig. 1E) 1.12× as long as high. Pronotum, moderately pubescent; lateral surface of pronotum with strong irregular reticulate rugose sculpture. Pronotum medially short; ratio of length of pronotum medially/laterally = 0.2. Pronotal plate indistinct dorsally.

Mesonotum. Mesoscutum (Fig. 1C) barely pubescent and with strong reticulate-rugose sculpture, the interspaces smooth and shining. Notauli somewhat obscured by the coarse sculpture, but visible; strongly convergent posteriorly; longitudinal median impression, not discernible. Anteroadmedian signa quite visible, extended back to near one half of mesoscutum; parapsidal signa distinct. Transscutal fissure narrow, sinuate. Mesoscutellum 1.2× as long as wide; about 0.7× as long as mesoscutum. Scutellar foveae (Fig. 1D) rounded, elongated posteriorly, about 0.5× as long as mesoscutellum, separated medially by a groove, the foveae are deep, mostly smooth anteriorly and crossed posteriorly by irregular transversal rugae, the intervals smooth, posterior margins indistinct. Mesoscutellum strongly coarsely rugose, with a deep and broad median longitudinal impression which makes the mesoscutellum strongly emarginate posteriorly (Fig. 1D); the emargination reaches anteriorly the scutellar foveae. In lateral view, the posterior emargination of mesoscutellum is seen as two, slightly curved upwards, horn-like projections. Mesoscutellum in lateral view with the posterodorsal extension of body of subaxillular strip short, not reaching one half of mesoscutellar height. Mesopleuron coarsely reticulate rugose, the rugae not as strong as in mesoscutum (Fig. 1E).

Metanotum. Metapectal-propodeal complex. Metapleural sulcus reaching posterior margin of mesopectus at about mid-height of metapectal-propodeal complex. Metascutellum rugose; metanotal trough smooth and pubescent. Median propodeal area (Fig. 1F) with some irregular strong longitudinal and transversal rugae; and densely pubescent; lateral propodeal carinae distinct, subparallel anteriorly and converging posteriorly.

Legs. Densely pubescent; femora and tibiae robust. Metatibia about as long as metatarsus; apical margin of metatarsomeres 1–4, with long strong erect setae. Metatarsal claws with strong triangular basal lobes or teeth.

Forewing (Fig. 4A), about as long as body, radial cell 3.2× longer than wide; open along anterior margin; areolet obsolete, obscured by infuscation. M and Cu1 veins nearly straight, not reaching wing margin. Rs+M not reaching basalis. First abscissa of radius (2r) slightly angled, not projected. Cu1 vein not branched in two veins. Apical margin with very short or obsolete hair fringe.

Metasoma (Fig. 3A, D), in dorsal view 1.6× as long as wide, in lateral view 1.2× as long as high. Second metasomal tergite covering about 0.7× length of metasoma. Anterior 2/3 smooth and shining; posterior one third with a band of micropunctures clearly visible; the punctate sculpture extended on subsequent tergites; ventral area of second metasomal tergite moderately pubescent. Projecting part of hypopygial spine moderately long (Fig. 3B); 4.6× as long as high in lateral view; laterally with long setae, longer than spine width, but not forming an apical patch.

Male (Figs 2A–F, 4B, D). Differs from the female as follows: smaller size, length 5.2 mm on average (n = 3). Body and wings almost completely black, except tarsomeres of anterior legs and apical segments of antennae. Antennae, legs and wings relatively longer. Antenna (Fig. 3E) with 14 segments. Antennal formula as: 0.24(0.15):0.13(0.15):0.6(0.11):0.39(0.13):0.35(0.14):0.35(0.14):0.32(0.12): 0.31(0.11):0.28(0.11): 0.27(0.1):0.27(0.1):0.27(0.1):0.26(0.1):0.24(0.1):0.23(0.08). F1 slightly curved and enlarged apically and flattened ventrally, 1.5× as long as F2; placodeal sensilla present in all the flagellomeres (Fig. 3F). Head 1.3× as wide as high; apical part of gena slightly expanded. Mesoscutellar impression not reaching the scutellar foveae (Fig. 2D). Scutellar foveae confluent, not separated by a sulcus. Forewing relatively longer 1.2× as long as body. Almost completely black, except the distal transversal clear band (Fig. 4B).

**Gall** (Fig. 5A–D). A large spindle-shaped gall with an elongated and narrow tip and base. The galls measure 100 × 25 mm on average. The surface of the gall is smooth, but some superficial longitudinal ridges are visible. The gall is monothalamic; the outer shell is thin, flexible and of fleshy consistency when it is fresh and becomes soft and light when it dries. They are light green without spots when they are fresh and light brown when they are dry. Internally (Fig. 5C), there is an oval larval cell in the centre of the gall (0.35 mm thick and 7 × 5 mm; n = 1). A spongy tissue occupies the entire space between the epidermis and the larval chamber, the outer shell is weakly attached to the internal spongy tissue when fresh and when the gall dries, the spongy tissue allows us to observe the radiant filaments, which extend from the larval chamber towards the internal walls of the galls (Fig. 5D). When it is dry, the gall is very fragile and can be easily crushed. At least half of the galls no longer showed spongy tissue when they were transferred to the laboratory. This caused the galls to collapse due to the fragility of the epidermis. Some of these collapsed galls presented internal modifications in the epidermis, probably caused by inquilines.

The galls develop on twigs of *Quercus zempoaltepecana* Trel. The gall closely resembles that of *Amphibolips durangensis* Nieves-Aldrey & Maldonado, 2012. However, the gall of *A. magnigalla* is distinguished by its larger size, which is at least 2× longer than that of *A. durangensis* and by its different internal structure, which is filled with less dense spongy tissue and radiant filaments (easily visible in the older galls).

#### Distribution.

*A. magnigalla* was found only in one site: Comaltepec (Oaxaca State, Mexico). The galls were relatively abundant on a single isolated tree, while we did not find galls on the nearby trees.

#### Biology.

Sexual generation. The galls were collected at the end of April and the insects emerged shortly thereafter, in early May. It seems that it is normal for many insects to feed on the tissue of this species. A detached gall was observed in a field, relatively far from the tree, probably carried by a bird.

### Amphibolips kinseyi

Taxon classificationAnimaliaHymenopteraCynipidae

Nieves-Aldrey & Castillejos-Lemus sp. nov.

4DA5E610-9FA0-5CE5-A336-FCF3F2C9314C

http://zoobank.org/5542112A-D80F-4FF1-BB9F-623D326833BC

**Figure 6.**
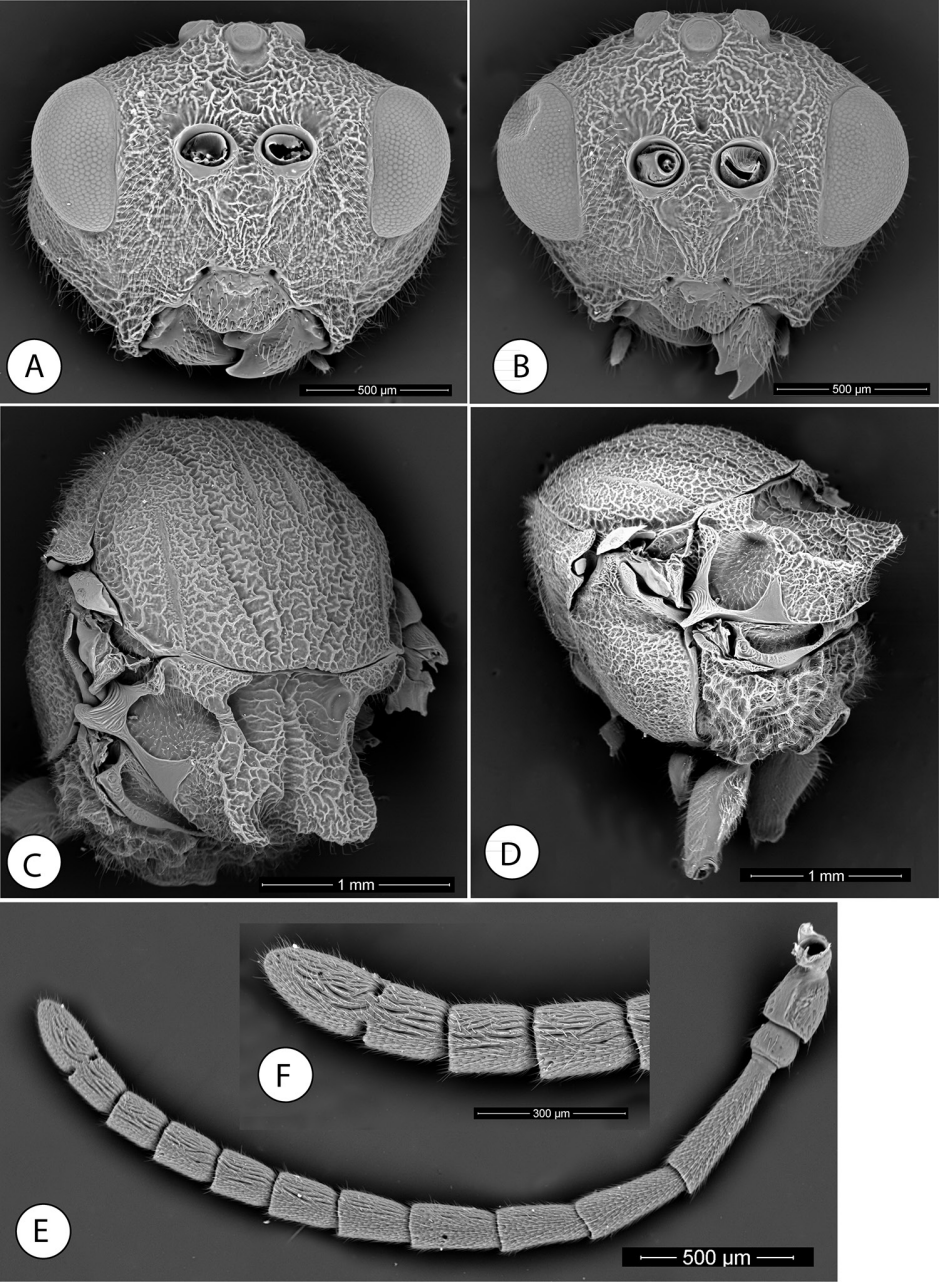
*Amphibolips kinseyi* sp. nov. **A** female head, anterior view **B** male head, anterior view **C–D** female mesosoma, dorsal and lateral view **E** female antenna **F** detail of last flagellomeres in female antenna.

**Figure 7.**
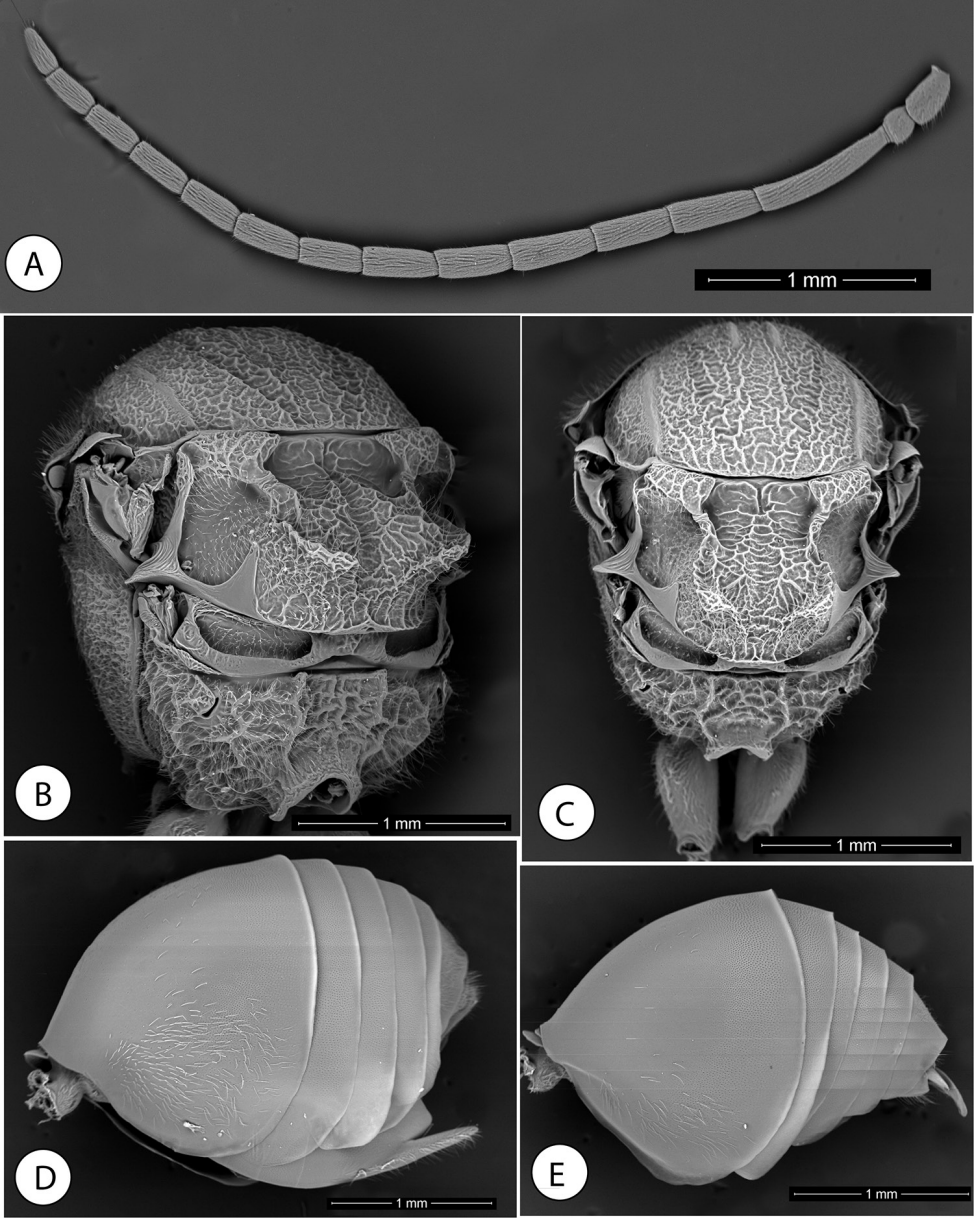
*Amphibolips kinseyi* sp. nov. **A** male antenna **B** female mesosoma, dorsolateral view **C** male mesosoma, dorsal view **D** female metasoma, lateral view **E** male metasoma, lateral view.

**Figure 8.**
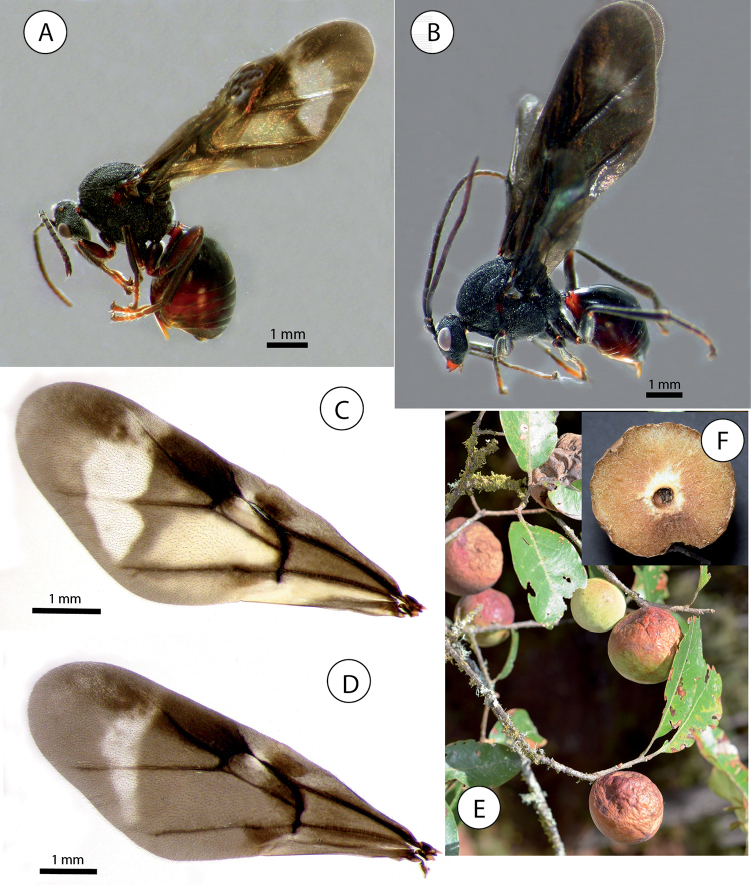
*Amphibolips kinseyi* sp. nov. **A** female habitus **B** male habitus **C** female forewing **D** male forewing **E** galls **F** section of a gall.

**Figure 9.**
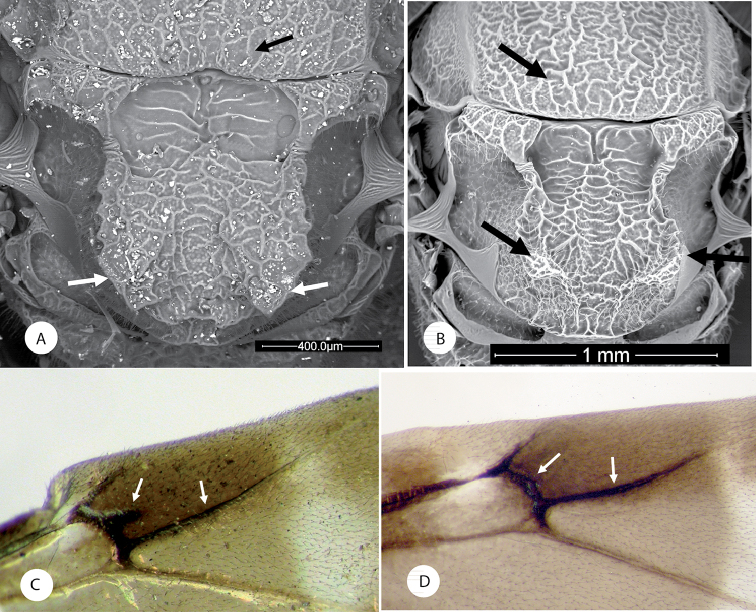
Comparison between *Amphibolips dampfi* and *A. kinseyi* sp. nov. **A** male mesoscutellum of *A. dampfi***B** male mesoscutellum of *A. kinseyi***C** radial cell of *A. dampfi***D** radial cell of *A. kinseyi*.

Figs 6 , 7 , 8 , 9B, D

#### Type material.

***Holotype***: 1♀ in the Museo Nacional de Ciencias Naturales (MNCN), Madrid, Spain, mounted (glued) on a card. Mexico, Oaxaca, Pozuelos, Ixtlán, 17°22.52'N, 96°26.88'W, ca. 3040 m alt., ex gall *Quercus zempoaltepecana*. Collected 21/04/2018; emerged 04/05/2018. D. Castillejos-Lemus leg. ***Paratypes***: 4♂ and 2♀, same data as that of holotype; 1♀ and 1♂ paratype dissected and mounted on a stub for SEM observation. Additional material in ethanol: 3♂ and 1♀ (in the collection of Castillejos-Lemus, Morelia, Mexico), 1♂ (in MNCN). Eighteen galls, one dissected (in the collection of Castillejos-Lemus and the Colección Nacional de Insectos-UNAM).

#### Etymology.

Named after Dr Alfred Kinsey, one of the most prominent cynipidologists and the pioneer of the study of *Amphibolips* in Mexico.

#### Diagnosis and comments.

*Amphibolips kinseyi* is very similar to *A. dampfi* Kinsey, 1937. We collected the new species in sites near where collections by A. Dampf were made (near Ixtlán, Oaxaca), the material of which was later described by Kinsey (Kinsey 1937). Both species share a strongly emarginate mesoscutellum and have a similar forewing colour pattern. However, after a close comparison with the male holotype, we found some diagnostic differences that allowed us to describe our specimens as different and new species. The forewings of the males of *A. dampfi* and *A. kinseyi* are similar, being predominantly black infuscate and have a reduced clear transversal band; however, in *A. dampfi*, the first radial abscissa (2r vein) is strongly angled and projected into the radial cell (Fig. 9C), while it is weakly angled and not projected in *A. kinseyi* (Fig. 9D). The postero-lateral projections of the mesoscutellum are more or less pointed or acute in the *A. dampfi* males (Fig. 9A), but are more rounded and flatter in the case of the new species (Fig. 9B). Additional distinguishing characters are given in the identification key provided herein. The female forewing of *A. dampfi* was not available for description, as it was apparently lost in the only female collected. However, the female forewing of this closely-allied new species is described here. Consistent with other *Amphibolips* species from Mexico, the female forewing is different from the male forewing. In this case, the female forewing has a clear transversal band, which is larger and more extended than that of the male (Fig. 8C).

#### Description.

Body length: 6.3 mm (n = 1) for females; 5.7 mm (n = 3) for males.

Female. Body predominantly black (Fig. 8A); head, except the red mandibles and the mesosoma, black; metasoma reddish postero-ventrally; antennal flagellum reddish in distal half; legs reddish except black basal part of coxae. Forewing (Fig. 8C) predominantly black infuscate, but much less infuscate above the cubital veins and below the M+Cu1 vein. There is a wide, clear, transversal band, which starts in the apex of the radial cell and extends towards the discoidal and cubital cells to the Cu1a vein but does not reach the latero-ventral margin of the forewing.

Head, in dorsal view 2.1× as wide as long; 0.8× as wide as mesosoma. POL 0.7× the OOL; lateral ocelli separated from inner margin of an eye for a distance of 3× the diameter of a lateral ocellus. Head in anterior view (Fig. 6A) 1.2× wider than high, gena slightly broadened behind eye. Vertex, frons, lower face and gena, with strong coarsely-rugose sculpture. A medial frontal pit visible followed by a sulcus addressed to the median ocellus. Face with two longitudinal carinae visible, extending from ventral margin of toruli to converge towards the space between the anterior tentorial pits; irradiating carinae from clypeus virtually absent; head moderately pubescent, except in vertex and frons. Clypeus more or less hexagonal, ventral margin strongly projecting over mandibles and weakly sinuate on anterior margin. Anterior tentorial pits well visible; epistomal sulcus and clypeo-pleurostomal lines visible. Gena slightly depressed basally and projected over the mandibles. Malar space 0.7× height of a compound eye. Toruli situated mid-height of compound eye; transfacial line 1.7× height of an eye; distance between antennal rim and compound eye 0.8× width of antennal socket including rim. Ocellar plate slightly raised.

Mouthparts (Fig. 6A), mandibles strong, exposed; with dense setae in base, right mandible with three teeth; left with two teeth.

Antenna (Fig. 6E), about one half as long as body length; with 13 antennomeres; 12 and 13 incompletely separated ventrally. Flagellum not broadening towards apex; with relatively long, erect setae. Relative length/width of antennal segments as: 0.28(0.16):0.16(0.16):0.52(0.16): 0.38(0.16):0.3(0.17):0.24(0.16):0.24(0.17):0.2(0.17):0.2(0.17):0.2(0.16):0.18(0.16):0.16(0.15):0.3(0.14). Scape slightly longer than wide, flattened and smooth ventrally. Pedicel, short, small, as long as wide, 0.5× as long as scape; F1 1.3× as long as F2, F11 2.3 times as long as wide, 2× as long as F10. Placodeal sensilla present on flagellomeres F3-F11, disposed in dense rows of 6–8 sensilla, only in half dorsal area of each flagellomere. Coeloconic and trichoidea sensilla are also present and visible (Fig. 6F).

Mesosoma in lateral view 1.3× as long as high. Pronotum, moderately pubescent; lateral surface of pronotum with strong irregular reticulate rugose sculpture (Fig. 6D). Pronotum medially short; ratio of length of pronotum medially/laterally = 0.2. Pronotal plate indistinct dorsally.

Mesonotum. Mesoscutum (Fig. 6C) barely pubescent and with strong coarse reticulate sculpture, the interspaces smooth and shining. Notauli shallow and crossed by the general sculpture, but well visible and almost complete; longitudinal median impression not visible. Anteroadmedian signa well marked, extended back to near one half of mesoscutum; parapsidal signa distinct. Transscutal fissure very narrow, sinuate. Mesoscutellum 1.3× as long as wide; about 0.5× as long as mesoscutum. Scutellar foveae ovoid elongated posteriorly, about 0.5× as long as mesoscutellum, separated medially by a deep groove, the foveae are deep, crossed by irregular transversal rugae, the intervals smooth, posterior margins shallowly indicated. Mesoscutellum strongly coarsely rugose, with a deep and broad median longitudinal impression which makes the mesoscutellum strongly emarginate posteriorly (Fig. 6C); the emargination reaches anteriorly the scutellar foveae. In lateral view, the posterior emargination of mesoscutellum appears as two rounded apically and slightly flat, curved upwards projections. In lateral view, the space between the mesoscutellar projections and the posterior limit of mesoscutellum is high. Axillula large, deep, heavily pubescent, with distinct margins. Mesoscutellum in lateral view with the posterodorsal extension of body of subaxillular strip long, nearly reaching upper margin of mesoscutellum. Mesopleuron coarsely reticulate rugose, the rugae not as strong as in mesoscutum.

Metanotum. Metapectal-propodeal complex. Metapleural sulcus obscured by the strong sculpture. Metascutellum weakly rugose; metanotal trough deep, smooth and pubescent. Median propodeal area with strong and coarse reticulate rugae; densely pubescent; lateral propodeal carinae distinct, subparallel anteriorly and converging posteriorly.

Legs. Densely pubescent; femora and tibiae robust. Femur 4× as long as wide; metatibia 1.6× as long as metatarsus; apical margin of metatarsomeres 1–4, with long strong erect setae. Metatarsal claws with strong triangular basal lobes or teeth.

Forewing (Fig. 8C), about 1.2× as long as body, radial cell 3.7× as long as wide; open along anterior margin; areolet very small, but visible. All veins heavily infuscate. M and Cu1 veins nearly straight, not reaching wing margin. Rs+M reaching basalis, well-marked. First abscissa of radius (2r) slightly angled, not projected. Cu1 vein not branched in two veins. Apical margin with very short or obsolete hair fringe.

Metasoma (Fig. 7D), in lateral view 1.3× as long as high. Second metasomal tergite covering about 0.6× length of metasoma. Anterior 2/3 smooth and shining; posterior one third with a band of micropunctures clearly visible; the punctate sculpture extended on subsequent tergites; ventral area of second metasomal tergite moderately pubescent, with a relatively dense patch of setae. Projecting part of hypopygial spine moderately long (Fig. 7D); about 5× as long as high in lateral view; laterally with long setae, longer than spine width, but not forming an apical tuft.

Male (Figs 6B, 7A, E, 8B, D). Differs from the female as follows: smaller size, length 5.7 mm on average (n = 3). Body and wings almost completely black, except the mandibles, metasoma ventrally, tarsomeres of legs and half of the apical flagellomeres of antennae which are more or less reddish (Fig. 8B). Antennae, legs and wings relatively longer. Antenna with 14 segments (Fig. 7A). Antennal formula (mean of four measured individuals) as: 0.29(0.18):0.15(0.16):0.69(0.16):0.46(0.15):0.40(0.15): 0.41(0.15):0.38(0.14):0.36(0.14):0.34(0.14):0.34(0.14):0.31(0.14):0.32(0.13):0.29(0.12):0.25(0.11):0.23(0.11). F1 slightly curved, weakly enlarged apically and flattened ventrally, 1.5× as long as F2; placodeal sensilla present in all the flagellomeres. Head 1.3× as wide as high; apical part of gena slightly expanded. Frontal pit distinct, prolonged by a groove towards median ocellus. Pair of frontal longitudinal carinae more convergent towards epistomal line (Fig. 6B). Projection of anterior margin of clypeus more incised. Forewing (Fig. 8D) relatively longer 1.3× as long as body. Almost completely black, except the distal transversal clear band that is much smaller and less extended.

#### Gall

(Fig. 8E, F). The gall is similar to the gall of *Amphibolips dampfi* described by Kinsey (1937). A moderate to large “oak apple”, irregularly spherical gall with spongy inner consistency. Some are slightly elongated towards the apex. The surface is slightly rough when intact, but may have more pronounced irregularities, which cause deformations on the surface or in the general shape. Monothalamic. They are light green without spots when they are fresh and light brown when they are dry. The epidermis is thin, at 0.4 mm thick; firmly attached to the internal spongy tissue when fresh; firm and brittle when dry. The consistency is relatively hard and fleshy when green and brittle when dry. Internally, the spongy tissue occupies the entire space between the epidermis and the larval chamber (Fig. 8F). Diameter of 30 mm and height of 31 mm on average (diameter of 16 to 44 mm and height of 18 to 51 mm; n = 18). Rigid and oval larval cell, 0.4 mm thick and 6.5 mm long × 5 mm in diameter on average (n = 2). Galls are formed on the twigs of *Quercus zempoaltepecana* Trel. Galls are relatively common in the study area.

#### Distribution.

Known only from the type locality along the route from Ixtlán to Tepanzacoalcos (Oaxaca State, Mexico).

#### Biology.

Sexual generation. The galls were collected in late April and the insects emerged shortly thereafter, in early May. It is normal to find galls deformed and/or attacked by inquilines and parasitoids; the deformed or attacked galls are usually relatively small.

### Amphibolips nigrialatus

Taxon classificationAnimaliaHymenopteraCynipidae

Nieves-Aldrey & Castillejos-Lemus sp. nov.

A58D9922-D7B9-5FBC-9FF0-52AE67FE8F57

http://zoobank.org/679F3F98-B166-4677-832C-AF7C6E0DB97C

**Figure 10.**
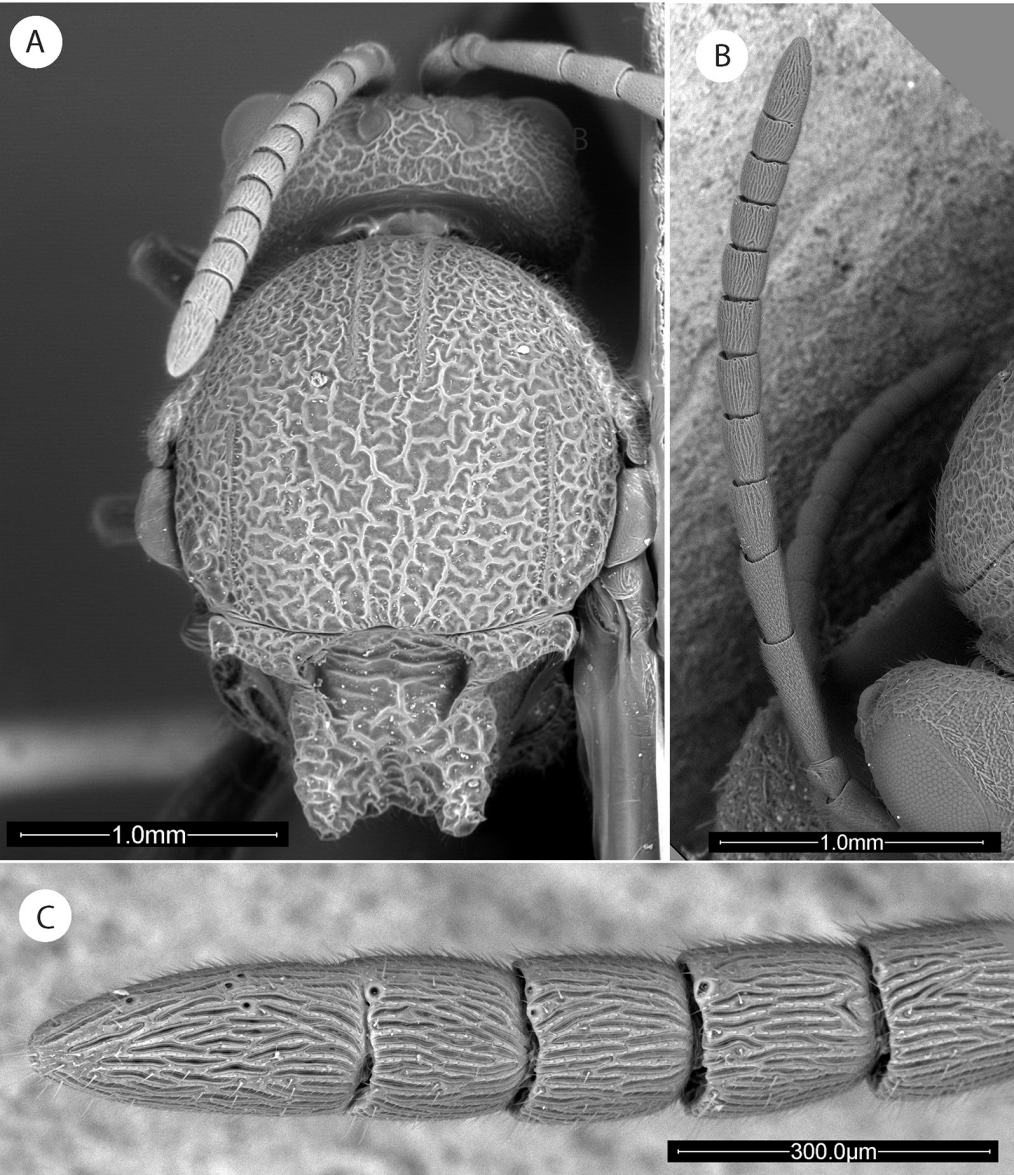
*Amphibolips nigrialatus* sp. nov., female. **A** head and mesosoma, dorsal view **B** antenna **C** detail of last flagellomeres.

**Figure 11.**
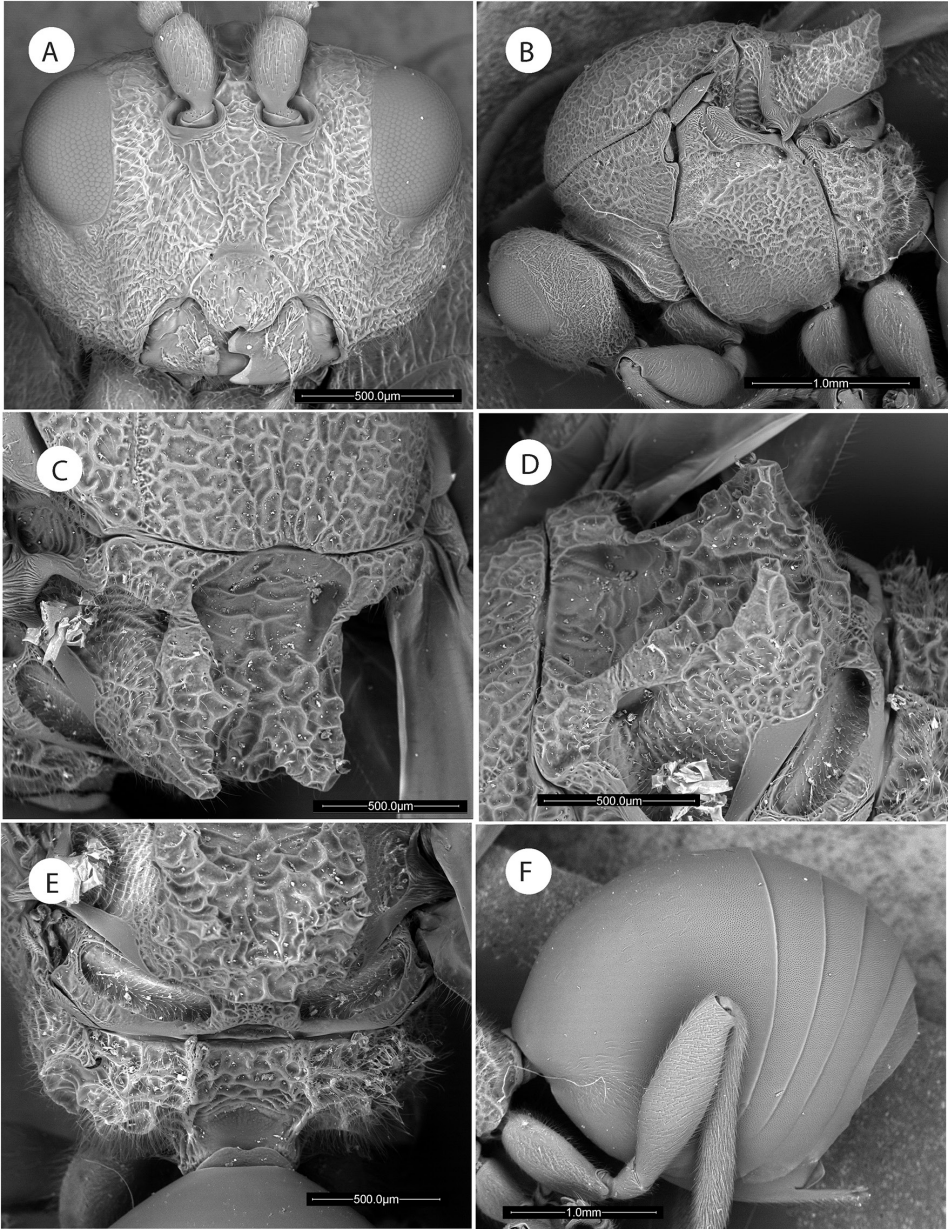
*Amphibolips nigrialatus* sp. nov., female. **A** head, anterior view **B** mesosoma, lateral view **C** mesoscutellum, dorsal view **D** mesoscutellum, lateral view **E** propodeum **F** metasoma, lateral view.

**Figure 12.**
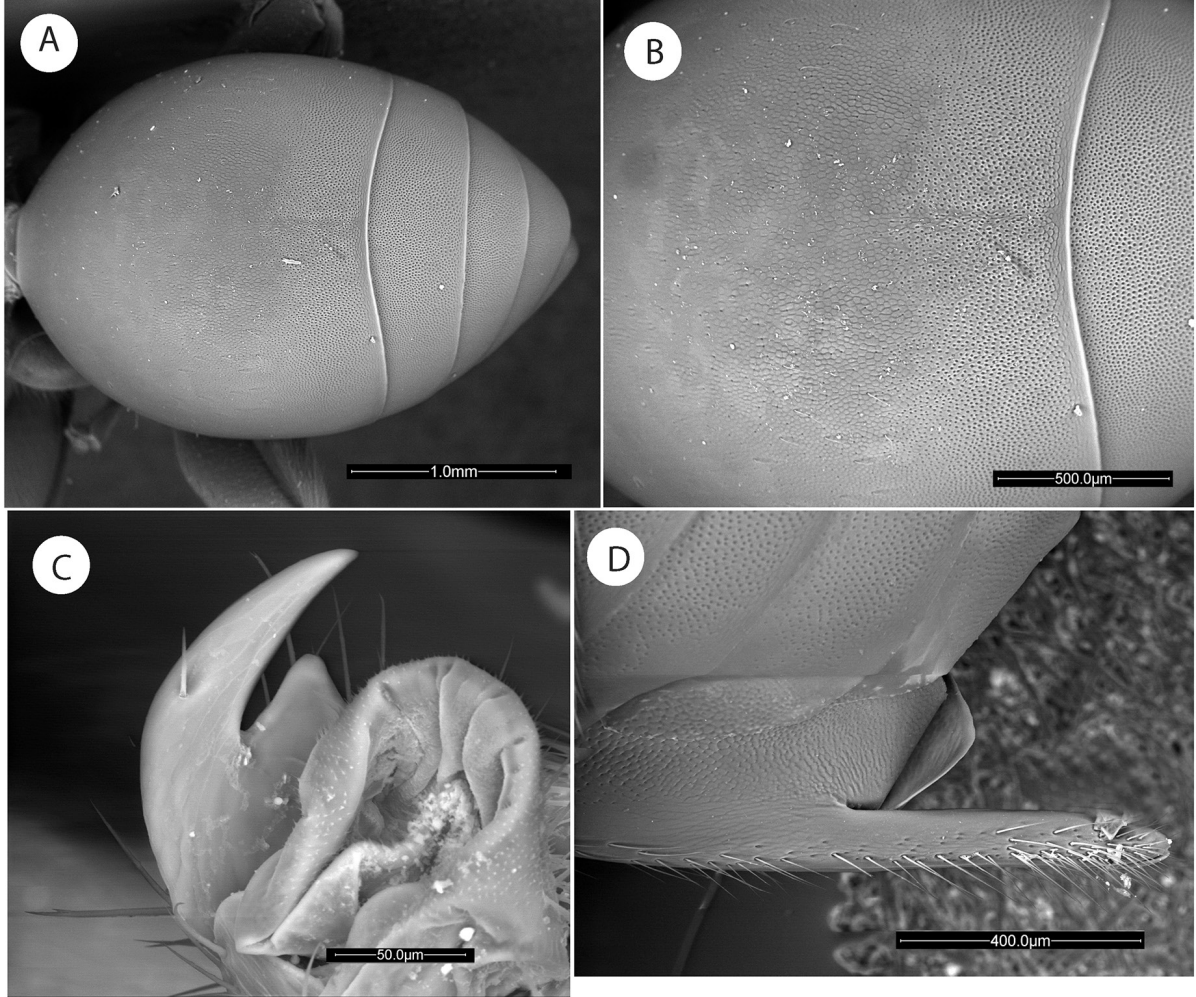
*Amphibolips nigrialatus* sp. nov., female. **A** metasoma, dorsal view **B** detail of sculpture **C** metatarsal claw **D** ventral spine of hypopygium, lateral view.

**Figure 13.**
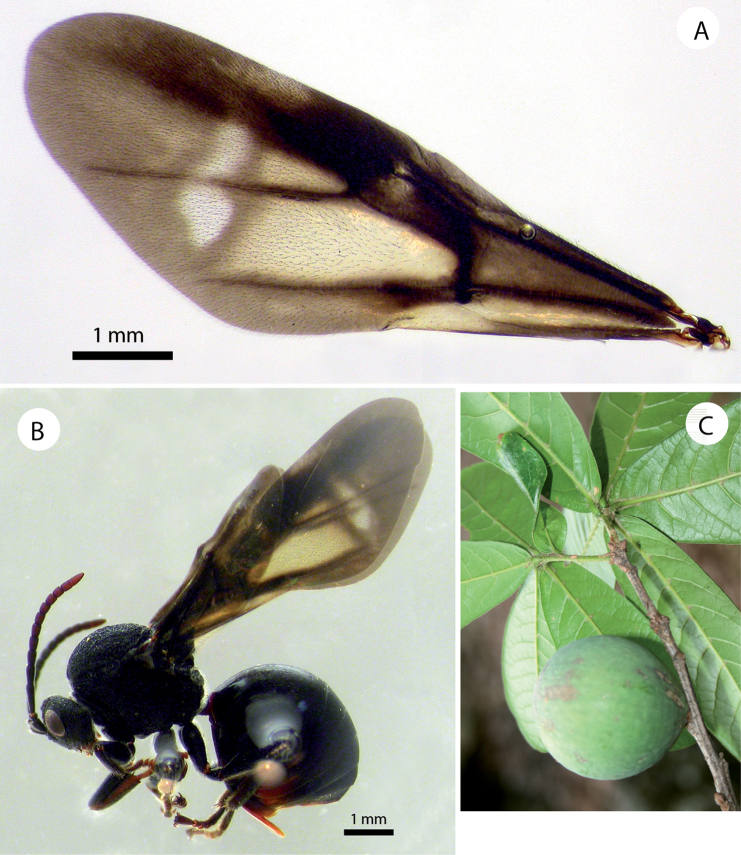
*Amphibolips nigrialatus* sp. nov., female. **A** forewing **B** habitus **C** gall on *Quercus sapotifolia* Liebm.

Figs 10 , 11 , 12 , 13

#### Type material.

**Holotype**: 1♀ in the Museo Nacional de Ciencias Naturales (MNCN), Madrid, Spain, mounted (glued) on a card. Mexico, Veracruz, Xico, in the Texolo waterfall, 19°24.11'N, 96°59.69'W, ca. 1170 m alt., ex gall *Quercus sapotifolia* Liebm. Collected 27/04/2008; emerged 04/2008. Nieves-Aldrey & Pascual leg.

#### Etymology.

Named after the smoky black forewing.

#### Diagnosis and comments.

*Amphibolips nigrialatus* is closely allied to *A. dampfi* Kinsey, 1937 and the new species *Amphibolips kinseyi*. Despite being based on a single female holotype, we found distinctive diagnostic characters that let us describe the specimen as belonging to a new species. The strongly-emarginate mesoscutellum relates the new species to *A. kinseyi* and *A. dampfi*, but in *A. nigrialatus*, the postero-lateral projections of the scutellum are pointed apically and curved upwards. Moreover, the scutellar foveae are very large in the new species, extending approximately one half of the length of the mesoscutellum, medially confluent and not separated by a carina or groove, while they are well separated by the mesoscutellar impression in the other two species. The forewing colour of *A. nigrialatus* is the darkest we have seen in females of *Amphibolips* species from Mexico and the black smoky colouration even extends to the costal and basal cells and below the cubital vein. In this last character, it resembles *A. castroviejoi* from Panama, but in this late species, the forewing area anterior to the transversal band is even darker (Fig. 15F), besides other distinguishing characters given in the identification key (transversal clear band larger, smooth scutellar foveae and visible notauli). The clear transversal band in the discoidal cell of forewing is very short and narrow in *A. nigrialatus*, measuring not more than one-fifth of the length of the radial cell (Fig. 13A), while in *A. kinseyi*, it is wider, measuring at least one-half the length of the radial cell (Fig. 8C). Additionally, the green spherical gall of the new species (*A. nigrialatus*) on *Quercus sapotifolia* is distinguishable from the galls of *A. dampfi* and *A. kinseyi* on *Q. ocoteifolia* Liebm. and *Q. zempoaltepecana* Trel., respectively.

#### Description.

Body length: 6.6 mm (n = 1) for the female.

Female. (Fig. 13B). Body almost completely black with the exception of the mandibles, the antennae apically, the metasoma ventrally, especially the hypopygium and parts of the legs, including the tarsi, which are reddish. Forewing predominantly black infuscate, except a large basal area delimited by the medial and cubital veins and another smaller area above the medial vein. A small clear transversal band, that starts below the radial cell and extends towards the medial vein, but does not reach the cubital vein, is present (Fig. 13A).

Head, in dorsal view (Fig. 10A) 2.2× as wide as long; narrower than mesosoma. OOL 1.4× POL 0.7× DOL; posterior ocelli separated from internal orbit of an eye by 2.2× its diameter. Head in anterior view (Fig. 11A) 1.3× as broad as high; gena slightly broadened behind eyes. Vertex, frons, lower face and gena, with coarse reticulate-rugose sculpture. Face with two longitudinal carinae, extending from ventral margin of toruli to converge towards the anterior tentorial pits; irradiating carinae from clypeus indistinct or absent; head moderately pubescent, except in vertex and frons. Clypeus more or less hexagonal, ventral margin strongly projecting over mandibles and moderately incised or sinuate on anterior margin. Anterior tentorial pits well visible; epistomal sulcus and clypeo-pleurostomal lines also distinct. Malar space 0.8× height of a compound eye. Toruli situated about mid-height of compound eye; transfacial line 1.7× height of an eye; distance between antennal rim and compound eye almost equal to the width of antennal socket including rim. Ocellar plate slightly raised.

Mouthparts (Fig. 11A), mandibles strong, exposed, with dense setae in base, right mandible with three teeth, left with two teeth.

Antenna (Fig. 10B), 0.6× as long as body length; with 13 antennomeres; 12 and 13 incompletely separated (Fig. 10C). Flagellum not broadening towards apex; with relatively long, erect setae. Antennal formula : 0.4(0.22):0.16(0.18):0.58(0.18):0.36(0.18):0.32(0.18):0.28(0.18):0.26(0.18):0.24(0.18): 0.23(0.18):0.2(0.18):0.2(0.18):0.18(0.18):0.34(0.16). Pedicel, short, 0.4× as long as scape and slightly broader than long. F1 1.6× as long as F2; F8–F10 as long as wide, F11 2.1× times as long as wide, 1.9× as long as F10. Placodeal sensilla present on flagellomeres F3–F11, disposed in dense rows of 8–10 sensilla, only in half dorsal area of each flagellomere.

Mesosoma in lateral view 1.1× as long as high. Pronotum, moderately pubescent; lateral surface of pronotum with strong irregular reticulate rugose sculpture (Fig. 11B). Pronotum medially short; ratio of length of pronotum medially/laterally = 0.23. Pronotal plate indistinct.

Mesonotum. Mesoscutum (Fig. 10A) barely pubescent and with strong coarse reticulate sculpture, the interspaces smooth and shining. Notauli indistinct, obscured by the coarse sculpture; more so in anterior one third of mesoscutum; longitudinal median impression indistinct. Anteroadmedian signa and parapsidal signa distinct. Transscutal fissure very narrow, sinuate. Mesoscutellum as long as wide; about 0.5× as long as mesoscutum. Scutellar foveae rounded transverse, about 0.5× as long as mesoscutellum, the scutellar foveae are confluent and not separated medially by a carina or groove; some transverse strong rugae visible with smooth and shining intervals. Mesoscutellum strongly coarsely rugose, with a deep and broad median longitudinal impression which makes the mesoscutellum strongly emarginate posteriorly (Fig. 10A); the emargination not reaching anteriorly the scutellar foveae. Postero-lateral projections of scutellum pointed apically and curved upwards (Fig. 11C, D). Mesopleuron coarsely reticulate rugose, the rugae not as strong as in mesoscutum (Fig. 11B). Mesoscutellum in lateral view with the posterodorsal extension of body of subaxillular strip short, not reaching one half of mesoscutellar upper margin.

Metanotum. Metapectal-propodeal complex. Metapleural sulcus distinct, reaching posterior margin of mesopectus at about mid-height of metapectal-propodeal complex. Metascutellum rugose; metanotal trough deep, smooth and pubescent. Median propodeal area with strong and coarse reticulate rugae; densely pubescent; lateral propodeal carinae distinct, subparallel anteriorly and converging posteriorly (Fig. 11E).

Legs. Densely pubescent; femora and tibiae robust. Metatibia about 1.7× as long as metatarsus. Metatarsal claws with strong triangular basal lobes or teeth (Fig. 12C).

Forewing (Fig. 13A), about 1.1× as long as body, radial cell 3.9× as long as wide; open along anterior margin; areolet absent. All veins heavily infuscate. M and Cu1 veins nearly straight, not reaching wing margin. Rs+M complete, reaching basalis. First abscissa of radius (2r) slightly angled, not projected into the radial cell. The two branches of the cubitalis vein are not interrupted by a gap. Apical margin with very short hair fringe.

Metasoma (Fig. 12A) in dorsal view 1.5× as long as wide; in lateral view 1.2× as long as high (Fig. 11F). Second metasomal tergite covering about 0.64× the length of metasoma. In dorsal view, anterior half of T2 smooth and somewhat shining, posterior half with two types of microsculpture clearly visible, first a series of slightly hexagonal cells and in the back micropunctures, both microstructures occupying the same proportion (Fig. 12B). In lateral view, anterior 2/3 smooth and somewhat shining; posterior one third with a band of micropunctures clearly visible. The punctate sculpture extended on subsequent tergites. Ventral area of second metasomal tergite moderately pubescent, with a relatively dense patch of setae. Projecting part of hypopygial spine long (Fig. 12D); about 6.3× as long as high in lateral view; laterally with long setae, longer than spine width, but not forming an apical tuft.

Male. Unknown.

#### Gall

(Fig. 13C). A regular, spherical, moderately-sized gall with a green colour when it is fresh. When dry, the gall acquires a rough and slightly elongated appearance and turns brown in colour. The galls measure on average 16.5 × 21.5 mm (diameter of 14 to 19 mm and length of 17 to 25 mm; n = 4). The gall is monothalamic. The outer shell is thin, flexible and of fleshy consistency when fresh and becomes rigid and hardly detachable from the parenchyma when dry. Internally, there is a spherical larval cell in the centre of the gall (5 × 5 mm; n = 1); a spongy tissue occupies the entire space between the epidermis and the larval chamber and is hardly separable from the larval chamber. When it is dry, the gall is moderately fragile.

On twigs of *Quercus sapotifolia* Liebm. Closely resembles that of *Amphibolips oaxacae* Nieves-Aldrey & Pascual, 2012, *A. michoacaensis* Nieves-Aldrey y Maldonado, 2012, *A. trizonata* Ashmead, 1896 and *Amphibolips kinseyi* sp. nov. However, the gall of *A. nigrialatus* differs in its size, which is approximately half that of the other species. The gall is similar to that of *A. murata* Weld, 1957, but not as rough when dry and to that of *A. quercusfuliginosa* Ashmead, 1885, from which it is impossible to differentiate according to the original description of the gall. Nonetheless, the adults are completely different.

#### Distribution.

Known only from the type locality in Veracruz State, Mexico.

#### Biology.

Presumably, a sexual generation. The gall was collected in late April and the insect emerged shortly afterwards.

**Figure 14.**
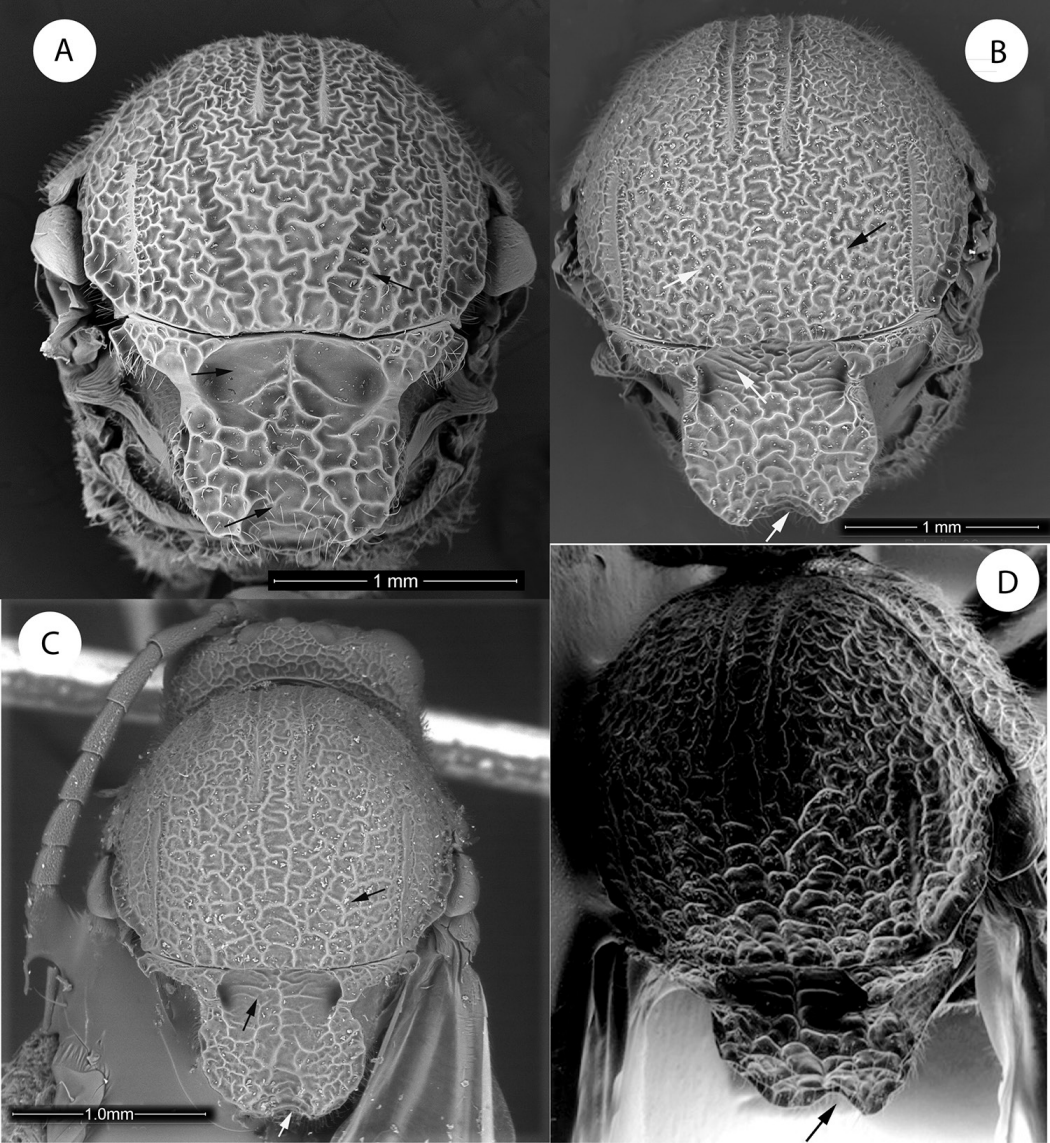
Details of mesoscutum and mesoscutellum (dorsal view) of *Amphibolips* species **A***Amphibolips castroviejoi* Medianero & Nieves-Aldrey **B***Amphibolips durangensis* Nieves-Aldrey & Maldonado **C***Amphibolips fusus* Kinsey (type) **D***Amphibolips cibriani* Pujade-Villar (last image taken from Pujade-Villar et al. 2018).

**Figure 15.**
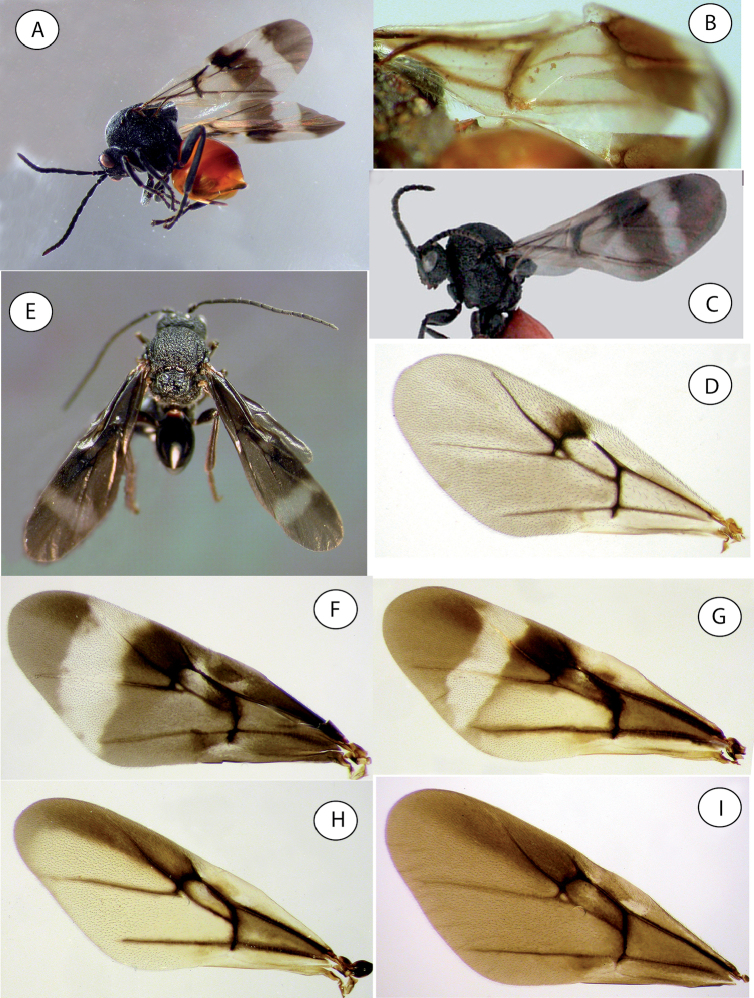
Details of wings and habitus of *Amphibolips* species **A** habitus of Amphibolips near fusus from Zacatecas **B** detail of forewing of *Amphibolips fusus* Kinsey (type) **C** habitus of *Amphibolips cibriani* Pujade-Villar (image taken from Pujade-Villar et al. 2018) **D** forewing of female *Amphibolips aliciae* Medianero & Nieves-Aldrey **E** habitus of male *Amphibolips castroviejoi* Medianero & Nieves-Aldrey **F** forewing of female *Amphibolips castroviejoi***G** forewing of female of *Amphibolips durangensis* Nieves-Aldrey & Maldonado **H** forewing female of *Amphibolips michoacaensis* Nieves-Aldrey & Pascual **I** forewing male of *Amphibolips michoacaensis*.

### Key to adult *Amphibolips* species of Mexico and Panama (species of the “*niger* complex” excluded). Modified from Nieves-Aldrey et al. (2012) for including the new species described

**Table:** 

1	Females. Antenna with 13–14 antennomeres; F1 not modified (Fig. 3C)	**2**
–	Males. Antenna with 15 antennomeres; F1 modified, flattened ventrally (Fig. 3E)	**9**
2	Forewing with a heavily-infuscate spot on the basal area of radial cell; remainder of the forewing, hyaline to only slightly infuscate (Fig. 15D)	***aliciae* Medianero & Nieves-Aldrey**
–	Forewing entirely infuscate, more heavily along a band on anterior margin of wing (Fig. 15G, H)	**3**
3	More heavily infuscate band along anterior margin of forewing with a clear transversal band on one-third apical part of radial cell which is more or less extended towards posterior margin of wing (Fig. 15F, G)	**4**
–	More heavily infuscate band along the anterior margin of the forewing, without a clear transversal band on apical part of radial cell extended towards posterior margin of wing. If there is a clear colourless spot apically on the radial cell, it does not extend below the radial cell (Fig. 15H)	^*^
4	Basal and first cubital cells colourless or only weakly infuscate prior to the heavily-infuscate basal half of the radial cell (Fig. 15A, B). Mesoscutellum weakly emarginate posteriorly (Fig. 14C). F1 1.2× as long as F2	***fusus* Kinsey** ^**^
–	Basal and first cubital cells as heavily infuscate as basal half of radial cell (Figs 4A, 15G). F1 1.4–1.5× as long as F2	**5**
5	Mesoscutellum slightly or moderately emarginate posteriorly (Fig. 14B). Forewing not as blackish infuscate; the clear transversal band relatively large and broad and extended to ventral margin of forewing	**6**
–	Mesoscutellum strongly emarginate posteriorly (usually V-shaped in dorsal view) (Figs 6C, 7B, 10A). Forewing darker, heavily infuscate and predominantly black; the clear transversal band usually smaller and not reaching ventral margin of forewing (Figs 8C, 13A); if reaching ventral margin (*A. castroviejoi*), then the scutellar foveae are smooth.	**7**
6	Mesoscutellum only slightly emarginate posteriorly (Fig. 14B). Clear transversal band relatively smaller and narrow (Fig. 15G)	***durangensis* Nieves-Aldrey & Maldonado**
–	Mesoscutellum more deeply emarginate posteriorly; postero-lateral projections of mesoscutellum moderately expanded (Fig. 1D). Non-infuscate clear forewing transversal band large and broad, almost reaching ventral margin of forewing; medially measuring approximately three-quarters of the length of the radial cell (Fig. 4A)	***magnigalla* sp. nov.**
7	Scutellar foveae smooth. Notauli visible (Fig. 14A). Basal area anteriorly the transversal clear band of forewing completely black; Costal cell infuscate; without less infuscate areas above the cubital veins and below the M+Cu1 vein (Fig. 15F). Transversal clear band relatively large, reaching ventrally the posterior margin of forewing (Fig. 15F)	***castroviejoi* Medianero & Nieves-Aldrey**
–	Scutellar foveae with carinate sculpture (Figs 2C, 10A). Notauli either visible (Fig. 6A) or almost invisible, hidden by sculpture on mesoscutum (Fig. 10A). Basal area anteriorly the transversal clear band of forewing with less infuscate basal areas, above the cubital veins and below the M+Cu1 vein (Figs 8C, 13A). Transversal clear band small and not reaching ventral margin of forewing; occupying about one third or less of the radial cell (Figs 8C, 13A)	**8**
8	Scutellar foveae large, extended approximately one-half of the length of mesoscutellum; medially confluent not separated by a carina or groove (Figs 11C, D). Posterolateral projections of scutellum pointed apically and curved upwards. Forewing strongly dark infuscate, the clear transversal band of the forewing small, short and narrow; medially not more than one-fifth the length of the radial cell (Fig. 13A).	***nigrialatus* sp. nov.**
–	Scutellar foveae not as large, extended approximately one-third of the length of the mesoscutellum and medially separated by the mesoscutellar impression (Figs 6C, D, 9A, B). Posterolateral projections of scutellum flat or less pointed apically, less upward curved. Clear forewing transversal band wider, medially about half of the radial cell	***kinseyi* sp. nov.**

Males

**Table:** 

9	Forewing with a heavily infuscate spot in the basal area of the radial cell; rest of the forewing only slightly infuscate	***aliciae* Medianero & Nieves-Aldrey**
–	Forewing entirely and heavily infuscate, with a transversal clear band or with a more infuscate longitudinal band on the anterior margin of the wing (Figs 4B, 8D, 15I)	**10**
10	Forewing with a clear transversal band on one-third of the apical part of the radial cell, which is more or less extended towards the posterior margin of the wing (Figs 4B, 8D)	**11**
–	Forewing without a clear transversal band on one-third of the apical part of the radial cell; usually with a more infuscate longitudinal band along dorsal margin of forewing; if there is a clear colourless spot apically on the radial cell, it does not extend below the radial cell (Fig. 15I)	^***^
11	Transversal clear band large; dorsally extended on 2/3 of apical area of radial cell and extended posteriorly to reach margin of the wing (Fig. 4B)	***magnigalla* sp. nov.**
–	Transversal clear band much more reduced in size; extended at most on one third of apical area of radial cell, and posteriorly not reaching the posterior margin of the wing (Figs 8D, 9C, 15E).	**12**
12	Scutellar foveae smooth. Transversal clear band relatively larger and more extended (Fig. 15C). Mesoscutellum moderately emarginate posteriorly	***castroviejoi* Medianero & Nieves-Aldrey**
–	Scutellar foveae sculptured. Transversal clear band very reduced in size (Figs 8D, 9C). Mesoscutellum strongly emarginate posteriorly (Fig. 9A, B), with a sharp horn projection observed in lateral view.	**13**
13	Posterolateral projections of the mesoscutellum acute pointed (Fig. 9A). Notauli hardly visible. Radial cell with first radial abscissa projected into the radial cell at tip of angle (Fig. 9C). F1 1.8× as long as F2	***dampfi* Kinsey**
–	Posterolateral projections of mesoscutellum flat and rounded apically (Fig. 9B). Notauli well indicated. Radial cell with first radial abscissa rounded or slightly angled, not projected into the radial cell at tip of angle (Fig. 9D). F1 1.6× as long as F2.	***kinseyi* sp. nov.**

## Discussion

The current study increases the number of *Amphibolips* in Mexico from 20 to 23. However, this number may rapidly increase since fieldwork is revealing new species that are still being studied and will eventually be published elsewhere.

The three species described herein present the typical diagnostic features of the *Amphibolips* species out of the “*niger*” group (Kinsey 1937, Melika et al. 2011, Nieves-Aldrey et al. 2012). We observed one morphological character, which seems to be shared only by *Amphibolips* species, namely, the absence of a gap or space between the two branches of the cubital vein of the forewing. A second not previously noticed character, present in the species from Mexico, is a pair of longitudinal carinae running from the ventral margin of the toruli to the anterior tentorial pits or the epistomal sulcus. However, this feature needs to be checked in all the *Amphibolips* species.

Considering that the complete life cycle of most species is unknown, it is difficult to propose a pattern of morphologies or phenologies for these species. In some cases, mistakes could be made, for example, some descriptions may pertain to different generations of currently-recognised species. Nieves-Aldrey et al. (2012) mentioned the difficulties in separating *Amphibolips* species (excluding those of the “*niger*” complex) based on external morphology of their galls, considering that some species are very similar (e.g. *A. nigrialatus* new species and *A. kinseyi* new species). In the same study, the question arises about the variability in a single *Amphibolips* species and the need to use new tools (specifically, molecular markers) that allow the clarification of the boundaries between species.

The new species described herein are very similar and share a set of similar morphological characteristics. The forewings are very dark and heavily black infuscate, but with a clear transversal band that is more or less extended in both sexes; the mesoscutellum is very deeply emarginate posteriorly, with the emargination reaching the scutellar foveae and almost dividing the mesoscutellum into two parts. Another shared feature of the three new species is that all are distributed in the States of Oaxaca and Veracruz in southern Mexico and none exceeds the Trans-Mexican volcanic belt. Their host oak species (*Quercus zempoaltepecana* and *Q. sapotifolia*) are similar in their affinity for slopes in humid climates near the Gulf of Mexico and their occurrence within tropical communities with many *Quercus* species. After studying large collections of *Amphibolips* collected across extensive areas of Mexico, we observed a distinct morphological pattern within the geographic distribution of the species, which is also confirmed in the species from the United States (unpublished observations). The more southern a species is distributed, the stronger and deeper its mesoscutellar emargination appears. These two patterns are consistent within a north-south distribution in Mexico.

The presence of four described species from the State of Oaxaca (*Amphibolips oaxacae* Nieves-Aldrey & Pascual, *A. dampfi* Kinsey, *A. magnigalla* sp. nov. and *A. kinseyi* sp. nov.) and three species from Panama (*A. castroviejoi* Medianero & Nieves-Aldrey, *A. aliciae* Medianero & Nieves-Aldrey and A. salicifoliae Medianero & Nieves-Aldrey) allows us to propose that *Amphibolips* must be present throughout Central America. Although they have not been cited from Chiapas, Mexico to Costa Rica, its presence in those geographic areas is likely, given that most host *Quercus* species recorded from south of Mexico and Panama are also present in Mesoamerica (Burger 1977, Breedlove 2001, Valencia 2004). Currently, broader work is being carried out to understand the relationships between the Mexican and Panamanian species of *Amphibolips*.

## Supplementary Material

XML Treatment for Amphibolips magnigalla

XML Treatment for Amphibolips kinseyi

XML Treatment for Amphibolips nigrialatus

## Footnotes

* To this couplet option run nine *Amphibolips* species from Mexico included in the above referred original key.

** To this couplet run also the recently described species *Amphibolips cibriani* Pujade-Villar. According the original description, we cannot find diagnostic morphological characters differentiating *A. cibriani* from *A. fusus* Kinsey (Figs 14C, D). The authors comparing incorrectly the species only with *A. castroviejoi* Medianero & Nieves-Aldrey and *A. durangensis* Nieves-Aldrey & Pascual, which have a different forewing colour pattern and run to the alternative option in this couplet (compare Fig. 15B, C). However, the galls of the two species are quite different: spindle-shaped in *A. fusus* while they are more or less spherical in *A. cibriani*.

*** To this couplet option run other nine *Amphibolips* species from Mexico included in the above referred original key.
